# Association of Primary Care Physicians’ Ambulatory Full-time Equivalencies With Time in the Electronic Health Record

**DOI:** 10.1001/jamanetworkopen.2023.20032

**Published:** 2023-06-21

**Authors:** Mark A. Micek, Adam Rule, Jeffrey J. Baltus, Brian Arndt

**Affiliations:** 1 Department of Medicine, Division of General Internal Medicine, University of Wisconsin School of Medicine and Public Health, Madison; 2 Information School, University of Wisconsin-Madison, Madison; 3 Department of Family Medicine and Community Health, University of Wisconsin School of Medicine and Public Health, Madison

## Abstract

This cross-sectional study examines whether primary care physicians (PCPs) in the clinic part-time have reduced electronic health record (EHR) time commensurate with their clinical hours.

## Introduction

Physicians with higher electronic health record (EHR) use report higher rates of burnout.^1^ Burnout is associated with leaving clinical practice and reducing clinical hours.^2,3^ Physicians may reduce clinical hours to reduce both EHR time and burnout. This study examines whether part-time physicians have reduced EHR time commensurate with their clinical hours.

## Methods

This cross-sectional study was approved by the University of Wisconsin–Madison (UW) institutional review board. This report follows the Strengthening the Reporting of Observational Studies in Epidemiology (STROBE) reporting guidelines for cross-sectional studies. Demographic, message, and EHR usage data were collected for UW Health primary care physicians (PCPs) with consistent ambulatory full-time equivalencies (aFTEs) across an 11-month period (May 2021-March 2022). We included FTE, scheduled hours, and EHR usage associated with all outpatient clinical work; inpatient FTE and EHR use were excluded. We categorized aFTE into 4 groups (≤0.40, 0.41-0.60, 0.61-0.80, and 0.81-1.00) and calculated 6 measures of EHR use for each PCP using data from our EHR (Epic Systems). Each EHR measure was normalized per week and per 8 hours of time scheduled with patients: total EHR time, inbox time, orders time, notes time, time outside scheduled hours on days with appointments (TOSH), and time on unscheduled days (TUSD). We evaluated the association of aFTE with EHR time using multivariable linear regression with log-transformation of EHR times because of their nonnormal distributions, adjusting for physician specialty, sex, and years at UW Health.

## Results

For the 118 physicians in this study (79 women [67%]), the median (IQR) aFTE was 0.67 (0.56-0.88), and 76 (64%) worked 10 years or longer at UW Health. Physician sex varied significantly by aFTE group, but not specialty or years at UW Health. Panel size, visit volume, and message volume were not significantly different between groups when normalized by aFTE or scheduled clinic hours (Table 1). Weekly total EHR, orders, and inbox time consistently decreased as aFTE decreased; notes, TOSH, and TUSD time did not follow a similar pattern (Table 2). Total EHR, notes, inbox, and TOSH time per 8 hours were all highest for physicians with 0.41 to 0.60 aFTE, and TUSD consistently increased as aFTE decreased (Table 2). Four model-adjusted measures of EHR use per 8 hours were significantly higher for PCPs with 0.41 to 0.60 aFTE vs those with 0.81 to 1.00 aFTE: total EHR (30.1%; 95% CI, 8.6%-55.8%; *P* = .005), inbox (60.0%; 95% CI, 29.5%-97.6%; *P* < .001), TOSH (103.3%; 95% CI, 29.5%-219.2%; *P* = .002), and TUSD (384.0%; 95% CI, 160.9%-797.7%; *P* < .001). Sensitivity analyses removing 13 physicians whose roles could require EHR use for nondirect patient care did not appreciably change the results.

**Table 1.** Characteristics of Study Participants by aFTE Group

Characteristic	Participants, No. (%)					*P* value^a^
	All (N = 118)	0.00-0.40 (n = 14)	0.41-0.60 (n = 36)	0.61-0.80 (n = 37)	0.81-1.00 (n = 31)	
Specialty						
Family medicine	48 (41)	5 (36)	14 (39)	15 (41)	14 (45)	.88
General internal medicine	47 (40)	7 (50)	13 (36)	14 (38)	13 (42)	
Pediatrics	23 (19)	2 (14)	9 (25)	8 (22)	4 (13)	
Sex						
Female	79 (67)	9 (64)	30 (83)	27 (73)	13 (42)	.003
Male	39 (33)	5 (36)	6 (17)	10 (27)	18 (58)	
Time at University of Wisconsin Health, y						
<5	15 (13)	0	5 (14)	4 (11)	6 (19)	.07
5-9.99	27 (23)	2 (14)	10 (28)	8 (22)	7 (23)	
10-14.99	29 (25)	1 (7)	12 (33)	9 (24)	7 (23)	
15-19.99	13 (11)	1 (7)	2 (6)	5 (14)	5 (16)	
≥20	34 (29)	10 (71)	7 (19)	11 (30)	6 (19)	
Workload						
aFTE, median (IQR)	0.67 (0.56-0.88)	0.27 (0.22-0.33)	0.56 (0.56-0.58)	0.70 (0.67-0.78)	0.93 (0.90-1.00)	<.001
Panel size, median (IQR), No. of patients^b^	1399 (1168-1892)	698 (526-810)	1214 (1103-1329)	1506 (1346-1745)	2060 (1897-2282)	<.001
Panel size/aFTE, median (IQR)^b^	2210 (1984-2417)	2434 (2128-2845)	2168 (1972-2421)	2136 (1945-2348)	2218 (2042-2357)	.31
Hours/week, median (IQR)	17.0 (13.2-21.4)	7.5 (6.1-8.4)	13.4 (12.5-14.7)	18.5 (16.7-20.1)	24.0 (22.5-25.9)	<.001
Visits/week, median (IQR)	30.7 (24.2-38.9)	14.1 (12.2-16.6)	25.1 (22.4-28.0)	31.9 (29.2-36.0)	41.9 (39.1-47.2)	<.001
Clinic days/week, median (IQR)	2.9 (2.5-3.5)	1.6 (1.4-1.9)	2.5 (2.2-2.7)	3.2 (2.7-3.4)	3.7 (3.4-4.3)	<.001
Visits/8 h scheduled clinic, median (IQR)	14.5 (13.4-15.4)	14.9 (13.4-17.5)	14.6 (13.8-15.4)	14.2 (13.3-14.9)	14.3 (13.6-15.1)	.50
Labor-intensive messages/week, median (IQR)^c^	114.6 (66.0-151.2)	59.4 (49.4-67.8)	98.3 (67.0-115.5)	124.6 (94.4-144.5)	163.1 (132.3-188.9)	<.001
Labor-intensive messages/8 h scheduled clinic, median (IQR)^c^	55.9 (43.5-68.0)	60.1 (53.7-74.1)	58.5 (37.7-73.5)	51.5 (44.9-59.4)	57.0 (43.5-63.2)	.34

Abbreviation: aFTE, ambulatory full-time equivalency.

^a^ *P* values (2-sided) were calculated from χ^2^ tests for count data and Kruskal-Wallis tests for continuous data across aFTE groups, with *P* < .05 considered significant.

^b^ Active panel was weighted by patient age, patient sex, payer, and relative value units associated with patient care.

^c^ Labor-intensive messages include telephone calls, patient-portal messages, laboratory results, medication management, and electronic consultations.

**Table 2.** Time in the EHR by Clinical aFTE

EHR time category and aFTE category	EHR time/week			EHR time/8 h		
	Raw median (IQR), min	Difference, % (95% CI)^a^	*P* value^a^	Raw median (IQR), min	Difference, % (95% CI)^a^	*P* value^a^
Total EHR time						
0.81-1.00	925 (661 to 1226)	0 [Reference]	NA	293 (234 to 408)	0 [Reference]	NA
0.61-0.80	767 (682 to 1119)	−8.3 (−23.2 to 9.4)	.33	352 (285 to 509)	19.9 (0.9 to 42.6)	.04
0.41-0.60	720 (578 to 866)	−23.8 (−36.7 to −8.4)	.004	418 (356 to 515)	30.1 (8.6 to 55.8)	.005
0.00-0.40	350 (291 to 444)	−62.2 (−70.4 to −51.9)	<.001	409 (328 to 477)	24.0 (−2.1 to 57.2)	.07
Time in notes						
0.81-1.00	260 (135 to 397)	0 [Reference]	NA	89 (42 to 127)	0 [Reference]	NA
0.61-0.80	239 (178 to 388)	−8.3 (−32.2 to 24.1)	.57	117 (74 to 161)	19.9 (−11.4 to 62.4)	.24
0.41-0.60	251 (192 to 331)	−24.3 (−44.8 to 3.6)	.08	144 (110 to 203)	29.2 (−5.7 to 77.1)	.11
0.00-0.40	86 (62 to 166)	−61.1 (−74.3 to −41.2)	<.001	119 (66 to 169)	27.8 (−15.5 to 93.4)	.24
Time in inbox						
0.81-1.00	162 (177 to 217)	0 [Reference]	NA	60 (36 to 72)	0 [Reference]	NA
0.61-0.80	145 (109 to 206)	1.2 (−17.9 to 24.6)	.91	66 (48 to 82)	32.3 (8.0 to 62.1)	.007
0.41-0.60	139 (119 to 189)	−6.3 (−24.6 to 16.4)	.55	83 (69 to 103)	60.0 (29.5 to 97.6)	<.001
0.00-0.40	85 (60 to 93)	−57.6 (−68.1 to −43.6)	<.001	76 (64 to 99)	39.4 (5.7 to 84.0)	.02
Time in orders						
0.81-1.00	154 (127 to 217)	0 [Reference]	NA	49 (42 to 74)	0 [Reference]	NA
0.61-0.80	123 (87 to 187)	−15.1 (−27.8 to 0.0)	.05	57 (42 to 76)	11.1 (−5.1 to 30.0)	.19
0.41-0.60	94 (77 to 124)	−37.8 (−47.5 to −26.2)	<.001	54 (43 to 70)	6.3 (−9.8 to 25.2)	.46
0.00-0.40	56 (47 to 70)	−68.4 (−74.7 to −60.5)	<.001	66 (47 to 80)	3.9 (−16.2 to 28.8)	.72
Time outside of scheduled hours on days with scheduled appointments						
0.81-1.00	165 (49 to 272)	0 [Reference]	NA	51 (16 to 85)	0 [Reference]	NA
0.61-0.80	143 (96 to 262)	24.7 (−19.6 to 93.3)	.32	64 (44 to 102)	63.1 (5.8 to 151.5)	.03
0.41-0.60	148 (106 to 229)	19.0 (−24.6 to 87.9)	.45	91 (54 to 135)	103.3 (29.5 to 219.2)	.002
0.00-0.40	67 (35 to 94)	−52.2 (−73.7 to −12.9)	.02	63 (53 to 99)	57.2 (−13.1 to 184.2)	.13
Time on unscheduled days						
0.81-1.00	47 (15 to 140)	0 [Reference]	NA	16 (6 to 43)	0 [Reference]	NA
0.61-0.80	123 (45 to 209)	137.5 (31.9 to 327.4)	.004	55 (20 to 95)	210.6 (71.6 to 462.1)	<.001
0.41-0.60	152 (89 to 224)	183.4 (53.7 to 422.6)	.001	88 (58 to 132)	384.0 (160.9 to 797.7)	<.001
0.00-0.40	98 (43 to 140)	78.4 (−20.1 to 298.5)	.16	132 (47 to 149)	486.1 (160.4 to 1219.3)	<.001

Abbreviations: aFTE, ambulatory full-time equivalency; EHR, electronic health record; NA, not applicable.

^a^ Model estimates and *P* values were calculated from multivariable linear regression of log-transformed time measures, adjusting for physician specialty, sex, and years at institution, with *P* < .05 considered significant.

## Discussion

This cross-sectional study found that physicians who worked part-time spent more time in the EHR per hour of clinic than their full-time peers, including more time outside scheduled appointments. This additional time was not explained by differences in panel size, visit volume, or message volume. Although order time was proportional to scheduled clinic hours, inbox time was not. Physicians with lower aFTE have more time outside clinic and may disproportionately fill it with inbox work that continues even on nonclinic days.

Physicians with lower aFTE may have lower rates of burnout,^4^ so the higher EHR time per 8 scheduled hours among lower aFTE physicians may not translate into higher rates of burnout. Although it sacrifices nonclinical time, more EHR work outside scheduled hours for lower aFTE physicians may allow them to feel caught up or allocate more time for panel management work not afforded to physicians with higher aFTE.^5^

Study limitations include small sample size, including administrative EHR use unrelated to patient care that may preferentially be done by lower aFTE physicians, and being a single-center study. Given the increasing EHR workload and shortage of PCPs in the US,^6^ future efforts should find ways to right-size workload for all aFTEs while allowing sufficient time for the inbox, proactive panel management, and quality improvement without affecting personal or nonclinical work time.

## Notes

Supplement.
Data Sharing Statement
