# Supplementary material for: Association of Primary Care Physicians’ Ambulatory Full-time Equivalencies With Time in the Electronic Health Record
Source: JAMA Netw Open. 2023 Jun 21;6(6):e2320032. doi: 10.1001/jamanetworkopen.2023.20032 (PMC10285567; doi:10.1001/jamanetworkopen.2023.20032)
Supplement: Supplement. — Data Sharing Statement [file jamanetwopen-e2320032-s001.pdf]

Data Sharing Statement

Micek. Association of Primary Care Physicians’ Ambulatory Full-time Equivalencies With Time in the Electronic Health Record. *JAMA Netw Open*. Published June 21, 2023. doi:10.1001/jamanetworkopen.2023.20032

Data
Data available: No

Additional Information
Explanation for why data not available: We do not have permission to share our partially-identified data
